# Early Recognition and Treatment of Malignant Hyperthermia in Pediatric Patient during Bronchoscopy

**DOI:** 10.1155/2020/6562896

**Published:** 2020-02-22

**Authors:** Warangkana Lapisatepun, Supawan Arkarattanakul

**Affiliations:** ^1^Department of Anesthesiology, Faculty of Medicine, Chiang Mai University, Chiang Mai, Thailand; ^2^Department of Anesthesiology, Faculty of Medicine, Mae Fah Luang University Hospital, Chiang Rai, Thailand

## Abstract

Malignant hyperthermia is a rare pharmacogenetic disorder triggered by depolarizing muscle relaxant and potent volatile anesthetic agents. An MH crisis is an emergency and life-threatening event requiring early recognition and prompt management. Dantrolene is the specific antagonist of MH. The authors report the case of a 9-year-old boy who underwent an emergency bronchoscopy to remove a foreign body and developed masseter rigidity after succinylcholine and sevoflurane exposure. The anesthesia team diagnosed an MH event, and the event was managed immediately with supportive treatment, dantrolene, being administered within 10 minutes. The patient survived and had a good outcome without any complications. We suggest that it is essential for anesthesia providers to recognize the need for intraoperative vigilance, prompt recognition, and treatment, and dantrolene sodium should be readily available in every hospital.

## 1. Introduction

Malignant hyperthermia is a rare anesthetic event triggered by succinylcholine and/or volatile anesthetics, resulting in a hypermetabolic state with a high mortality rate of 80–90% [1]. The incidence of MH is approximately 1 per 15,000 in pediatric patients if left untreated [2]. Dantrolene sodium is the specific treatment of MH, and the early diagnosis and prompt management with dantrolene leads to a decrease in the mortality rate to 5% [3]. This case study reports on the early diagnosis and successful treatment of MH in a pediatric patient scheduled for an emergency bronchoscopy to remove a foreign body.

## 2. Case Report

A healthy 9-year-old boy, of body weight 28 kg, was admitted to the university hospital with a foreign body (pin) in the left main bronchus without any signs of airway obstruction. He underwent an emergency bronchoscopy to remove the foreign body. The preanesthetic routine examination was normal. The patient had no significant medical conditions and no known medical allergies. He had never undergone general anesthesia, and his family had no familial history of unexplained death under anesthesia, anesthetic complications, or neuromuscular disease.

The patient received general anesthesia induced by propofol, 60 mg, and succinylcholine, 30 mg, given intravenously. He was ventilated with 100% oxygen and sevoflurane. After the onset of succinylcholine administration, the ENT surgeon tried to insert bronchoscope, but the child's mouth could not be opened. The anesthesiologist returned to ventilate with 100% oxygen and gave a small additional dose of 15 mg of succinylcholine. However, the masseter muscle rigidity in the patient continued. The trend of end tidal carbon dioxide rose from 30 mmHg to 55 mmHg, body temperature to 39.5°C, heart rate to 140–160 beats/minute, and blood pressure to 130/75 mmHg. Oxygen saturation was 100%. All of these symptoms in this patient were strong indications of malignant hyperthermia. The arterial blood gas was examined after the onset of symptoms, and the following results were found: pH 7.21, carbon dioxide partial pressure 63 mmHg (with appropriately controlled ventilation), oxygen partial pressure 245 mmHg, serum potassium 5.5 mmol/l, bicarbonate 25.2 mmol/l, BE 2.7 mmol/l, and hematocrit 33%. Five minutes after induction of anesthesia we recognized the early signs of malignant hyperthermia.

The anesthesiologist discontinued sevoflurane and hyperventilated with 100% oxygen, called for backup, changed to a new anesthesia machine prepared for a malignant hyperthermia patient, and informed the ENT surgeon. Anesthesia was maintained with total intravenous anesthesia (TIVA) with propofol infusion and mask ventilation with 100% oxygen. Cold normal saline was infused intravenously, and ice packs were applied to the entire body surface. Within approximately 10 minutes after the start of symptoms, dantrolene (20 mg vial of dantrolene in 60 ml sterile water) was given as a loading bolus of 2.5 mg/kg intravenously, and then 1 mg/kg IV every 6 hours until the signs and symptoms of MH subsided. After the first dose, the patient had an adequate clinical response, the trend of ETCO_2_ decreased to 38 mmHg, heart rate to 90–100 beats/minute, and blood pressure to 110/50 mmHg. All symptoms including masseter muscle rigidity, hyperthermia, and tachycardia were resolved. The ENT surgical team was informed regarding the condition of the patient. The surgical team decided to abort the endoscopic procedure. The patient was not intubated and was transferred to the pediatric intensive care unit (PICU) for further and supportive care.

After being admitted to the PICU, the patient was monitored closely for the signs of malignant hyperthermia and rhabdomyolysis. On postoperative day 1, the urine had a brown color, the serum creatinine kinase was still elevated at 9530 IU/L, serum potassium 5 mEq/L, and LDH 358 U/L. The patient received aggressive intravenous fluids to treat rhabdomyolysis. His medical condition improved, and dantrolene was discontinued on postoperative day 2. The patient came back to the operating room for a bronchoscopy to remove the foreign body on postoperative day 2. The anesthetic plan was total intravenous anesthesia (TIVA) induced with propofol, fentanyl, and atracurium and ventilation with 50% oxygen. During the operation, the patient's condition was stable with no signs of malignant hyperthermia. The surgical procedure was performed successfully, and the pin was removed from the left main bronchus. The patient was subsequently discharged home in a stable condition on postoperative day 4, the serum creatinine kinase having decreased to 855 IU/L. The anesthesia team counselled the patient's family members about this situation and the need to report this condition in case of future exposure of general anesthesia. This anesthetic event has been noted in the hospital medical records for future reference.

## 3. Discussion

Malignant hyperthermia (MH) is a life-threatening, rare, inherited skeletal muscle disorder with an incidence during anesthesia ranging from 1 : 10,000 to 250,000 [1, 4]. The clinical presentation of MH has hypermetabolic features that are triggered by the depolarizing muscle relaxant (succinylcholine) and potent inhalation anesthetics and is related to the uncontrolled release of intercellular calcium from the skeletal muscle sarcoplasmic reticulum. It results in an increase in heart rate, core body temperature, and end-tidal CO_2_ (ETCO_2_), tachypnea, and acidosis. An additional common sign in pediatric patients who received succinylcholine is masseter muscle rigidity.

Masseter muscle rigidity (MMR) referred to as “jaw of steel” results in reduced opening of the mouth and stiffness of jaw muscles. In certain instances, the other causes of jaw rigidity need to be differentiated from MMR, for example, temporomandibular joint dysfunction (TM joint dysfunction), pain-induced trismus, and myotonic syndrome. The underdosing of succinylcholine administration could be a cause of incorrect diagnosis of MMR [5, 6].

Masseter muscle rigidity (MMR) was the early indicator of malignant hyperthermia in patients of all ages, especially in pediatric patients; however, the incidence was less than 1 : 100, in those receiving succinylcholine during volatile anesthesia [7, 8]. In this patient, MMR was the earliest sign and was associated with hypermetabolic reaction. Therefore, malignant hyperthermia was the most likely diagnosis. In a similar case, some authors have reported a 4-year-old child who presented with succinylcholine-induced masseter muscle rigidity during emergency bronchoscopic removal of a tracheal foreign body [9].

Succinylcholine has been associated with the adverse effect of masseter muscle rigidity even though the child was not at risk of MH [10]. Isolated masseter muscle rigidity is not a pathognomonic sign of malignant hyperthermia [6, 11, 12], and MMR only occurs rarely after the administration of a nondepolarizing muscle relaxant [13–15]. It has been documented that the first dose of succinylcholine administration can cause a decrease in heart rate but not reach the bradycardia level; however, repeated doses of succinylcholine without pre-emptive atropine administration can result in significant bradycardia [16].

A clinical grading scale developed by Larach and colleagues is used to predict and diagnose MH. This scoring system is based on clinical manifestations and laboratory investigations including muscle rigidity and breakdown, respiratory acidosis, increased temperature, cardiac involvement, family history of MH, and other indicators. A score ≥50 suggests an almost certain case of MH [17], and our patient had an MH score of 63. The severity of MH may depend on the dosage given of the triggering agents [18].

Dantrolene sodium is the specific treatment in MH patients. It inhibits the dihydropyridine receptors in the and reduces calcium release from the sarcoplasmic reticulum. After dantrolene was introduced, the mortality rate decreased from 80% to 1.4% in North America [19]. In addition, supportive treatment is important, including discontinuing triggering agents, beginning active cooling techniques if hyperthermia has appeared, changing the breathing circuits, and increasing minute ventilation with 100% oxygen at high fresh gas flow to remove triggering agents. The Malignant Hyperthermia Association of the United States suggests that dantrolene must be available for administration within 10 minutes in anesthetizing locations where MH-triggering agents are used [20].

Dantrolene sodium is not available in some hospitals. Various authors have reported the survival of MH cases in pediatric patients without dantrolene administration, with early recognition and appropriate treatment leading to good outcomes in the event of MH [21, 22]. The prognosis of MH depends on early awareness and immediate appropriate management [23, 24]. In Thailand, not all anesthesia locations are fully stocked with dantrolene; however tertiary medical care centers are fully stocked with dantrolene. Our hospital is also a medical school and was fully stocked with dantrolene, so it was possible for this patient to receive proper management and dantrolene at the pertinent time. However, many hospitals in Thailand do not have an adequate supply of dantrolene, so the proper supportive management is essential for an MH patient's survival.

## 4. Conclusion

Early recognition of symptoms and appropriate treatment are the key to successful management of MH. Anesthesia providers should be aware of the symptoms and vigilant in patients with suspected MH. Prompt management is critical. Dantrolene sodium is the gold standard of MH treatment and should be available in all anesthesia locations. Family history and genetic counselling are the vital parts of MH management.
